# Association Between Motor Competence, Physical Fitness, and Academic Achievement in Physical Education in 13- to 16-Year-Old School Children

**DOI:** 10.3389/fspor.2021.774669

**Published:** 2022-01-20

**Authors:** Ruben Vist Hagen, Håvard Lorås, Hermundur Sigmundsson, Monika Haga

**Affiliations:** ^1^Department of Teacher Education, Norwegian University of Science and Technology, Trondheim, Norway; ^2^Department of Psychology, Norwegian University of Science and Technology, Trondheim, Norway

**Keywords:** lower secondary school, adolescents, movement proficiency, motor skills, physical capacity, grading

## Abstract

In physical education (PE), both assessment practices and choice of teaching content indicate that pupil-related factors such as motor competence and physical fitness potentially influence pupils' academic achievement in PE. However, neither of these factors are explicitly expressed as assessment criteria in the Norwegian PE-curriculum. Hence, the aim of the current study was to investigate potential differences in motor competence and physical fitness between pupils with different academic achievements in PE. Forty-five boys and 31 girls (*N* = 76) from grades 8 to 10 in the lower secondary school participated in this study. In addition to collecting pupils' final grade in PE, as a proxy for academic achievement, they were assessed on the Test of Motor Competence (placing bricks, building bricks, heal-to-toe-walking, and walking/running in slopes) and four task items from the Test of Physical fitness (standing broad jump, pushing medicine ball, running 20 m as fast as possible, and reduced Cooper test). In the main analysis, the total score for motor competence and physical fitness, as well as their respective task items, were compared between pupils with different grades (i.e., 3 or 4, 5, 6). A one-way ANOVA revealed neither significant difference between pupils with different grades in overall motor competence (total score) nor the respective task items for fine motor assessment or the gross motor task heal-to-toe-walking. However, there was a large difference between pupils with different grades in performance of the gross motor task walking/running in slopes, where pupils receiving a grade of 6 and 5 completed the task significantly faster compared to their peers with a grade of 3 or 4. Furthermore, a one-way ANOVA indicated moderate-to-large differences between pupils with different grades in the total score of physical fitness and performance in three of the four respective task items (i.e., standing broad jump, running 20 m as fast as possible, and reduced Cooper test). Pupils with a grade of 6 had a significantly greater total score of physical fitness and jumped longer compared to pupils with a grade of 5 and 3 or 4. Additionally, pupils with a grade of 6 ran significantly faster as opposed to pupils with a grade of 3 or 4. Performance on the reduced Cooper test was significantly different between all groups of grades, with the distance covered being progressively increased from pupils receiving a grade of 3 or 4 to 6, respectively. These results indicate that physical fitness levels and one component of motor competence may influence pupils' academic achievement in PE. Since neither certain levels of gross motor competence nor physical fitness are explicitly stated as assessment criteria in the Norwegian PE curriculum, these findings may indicate a lack of alignment between PE-teachers' assessment practice and the curriculum's intentions. It is argued that PE-teachers should be aware of how these individual constraints may influence pupils' academic achievement in PE so that all pupils are given equal opportunities to meet the described learning outcomes.

## Introduction

As a measure of academic achievement, the grade in Physical education (PE) should reflect pupils' attained level of competence (e.g., knowledge and skills) following the formulated learning outcomes expressed in the curriculum (LOVDATA, 2006a). In a Norwegian context, it is of importance that the grade in PE reflects a fair and objective assessment procedure as it functions as a selection instrument in the progress toward higher education and/or employment when, typically, summated with grades from other subjects (Leirhaug, 2016). However, pupils' achievement level in PE may be influenced by multiple constraints outside of that embodied within the curriculum, and certain pupils may have an advantage in this regard. For instance, according to the international literature, there is a prevailing sport- and exercise discourse within PE, which may shape and influence what PE-teachers consider as valuable pupil-related factors in relation to achievement (Redelius et al., 2009; Kirk, 2010; López-Pastor et al., 2013; Aasland and Engelsrud, 2017).

In the Norwegian PE curriculum, pupils in lower secondary school (13–16 years old) should develop their knowledge and skills within three main subject areas: sports activities (i.e., team and individual sports, dance, swimming and, alternative physical activities), outdoor life (i.e., skills and knowledge regarding how to make use of, and conduct and orient oneself in nature), and exercise and lifestyle (The Norwegian Directorate for Education Training, 2015a). For each of these subject areas, the PE-teachers' assessment in the subject should be based on the associated learning outcomes, entailing the pupils' ability to train, practice, and carry out different movement activities, as well as being able to explain and reflect around physical activity, body, and health-related issues (Aasland et al., 2020). In addition to the curriculum's formulated learning outcomes, pupils' effort constitutes the basis for assessment and grading in Norwegian PE (LOVDATA, 2006a). This is understood as pupils trying to solve PE-specific academic challenges, as well as challenging their own physical capacity to the best of their ability. Long-term continued practice with sustained effort, even if it does not necessarily result in improved performance or skill development, should positively impact pupils' academic achievement in PE (The Norwegian Directorate for Education Training, 2015b). Furthermore, learning outcomes formulated in the curriculum take into consideration pupils' individual prerequisites (e.g., physical precondition and motor skill levels) in the assessment of their achievement level (The Norwegian Directorate for Education and Training, 2015a). In summary, all pupils should have equal opportunities to achieve a top grade in the subject, despite any physical limitations. However, in Norway, PE-teachers are those primarily responsible for assessment and grading in the subject, being provided with few formalized regulations or specific criteria to guide their assessment and grading practice. Previous studies suggested that Norwegian, Swedish, and Dutch PE-teachers' assessment practice may not necessarily reference the described learning outcomes from the national curriculum (Annerstedt and Larsson, 2010; Arnesen et al., 2013; Leirhaug and MacPhail, 2015; Borghouts et al., 2017) and that the teachers may not always be able to express their own internalized criteria (Svenneberg et al., 2014). Hence, there is potential for subjective interpretations of the curriculum and assessment procedures which may have implications as to what pupil-related factors are deemed valuable in relation to academic achievement in PE.

In similarity with many other western countries, the sport and exercise perspective is evident within the Norwegian PE context (Aasland et al., 2017, 2020; Erdvik, 2020), which can shape and influence teaching content, methodology, and assessment practices. According to teachers' assessment practices, findings in the literature suggest that pupils need to perform well in sport-related skills and have a certain physical fitness level to achieve top grades in PE (Redelius et al., 2009; Hay and Macdonald, 2010; Prøitz and Borgen, 2010; Aasland et al., 2020). In this regard, both pupils' motor competence and physical fitness may act as relevant individual constraints to investigate. In a constraints-led perspective, pupils' academic achievement (i.e., grade) could be viewed as a result of the dynamical interaction between individuals (i.e., physical fitness, motor competence), task (e.g., teaching content), and environmental (e.g., the school) constraints acting upon teachers' assessment and grading (Newell, 1986; Davids et al., 2008).

Motor competence can be defined as an individual's level of performance in executing different motor actions, encompassing the coordination of both fine (e.g., manual dexterity) and gross motor skills (e.g., static and dynamic balance) (Henderson and Sugden, 1992). As such, motor competence can be understood as the degree of skilled performance in a wide range of motor tasks (including jumping, running, catching, throwing, kicking, and dribbling) as well as the movement quality, coordination, and control underlying a particular motor outcome (Burton and Miller, 1998; Utesch and Bardid, 2019). As a result, individual differences in motor competence may be reflected in pupils' academic achievement level in the subject when PE-teachers seem to place value on their ability to perform sport-related skills in relation to assessment (Aasland et al., 2020). Motor competence is also positively associated with physical activity levels and participation in organized activities in youth populations, in which children with higher levels of motor competence are more likely involved in sports (Fransen et al., 2014; Holfelder and Schott, 2014). As such, motor competence may indirectly influence achievement in PE through mediating the involvement in physical activity and sports outside of the school context where pupils experience movement activities in line with what is taught in PE (Moen et al., 2018).

Physical fitness, as inherent and achieved attributes, can be defined as an individual's capacity to execute physical activity or exercise (Caspersen et al., 1985; Ortega et al., 2008). Typically, assessment of physical fitness addresses components of force production, speed, strength, and endurance (Gallahue and Ozmun, 2006; Haga, 2009). National education policies in both Norway and Sweden have argued that the grade should not reflect pupils' physical fitness levels and that fitness testing in a norm-referenced assessment manner may be a direct violation of the regulations of the education act, which states that all pupils are entitled to equal opportunities within the subject (LOVDATA, 2006a; Redelius et al., 2009; The Norwegian Directorate for Education and Training, 2015b). However, evaluation and testing of physical fitness are used as part of teachers' assessment procedure, both in an international and a Norwegian context (Cale and Harris, 2009; Prøitz and Borgen, 2010; López-Pastor et al., 2013; Cale et al., 2014; Moen et al., 2018; Alfrey and Gard, 2019; Aasland et al., 2020; Marmeleira et al., 2020), and pupils' physical fitness levels probably influence their academic achievement in PE. Additionally, when it is suggested that Norwegian PE-teachers understand and interpret the effort criterion as pupils' ability to work physically hard, evident through their visible behavior (Aasland and Engelsrud, 2017), and teaching content being dominated by sport activities and fitness training (Moen et al., 2018), such factors may indirectly provide advantages to more physically fit pupils in terms of achievement in PE.

Both developments of motor competence and physical fitness are to varying degrees influenced by experience, growth, and maturational processes (Malina et al., 2004; Sigmundsson et al., 2017; Adolph and Hoch, 2019). Particularly, individual differences in timing and tempo of maturation become more pronounced during the adolescent growth spurt (Malina et al., 2004), a time associated with pupils attending lower secondary school (13–16 years old). As a result, differences in physical attributes and performance can be heightened during this period (Baxter-Jones, 1995; Philippaerts et al., 2006), which could be considered problematic when it seems PE teachers place value on sport-related skills and physical fitness in their assessment. Consequently, time spent in school (i.e., older pupils) may influence the grade they receive in PE (Cobley et al., 2009). With the adolescent growth spurt, gender-related differences also become more distinct. Boys tend to accelerate in motor- and fitness performance, surpassing the girls who improve slightly or level off (Malina et al., 2004). This might explain findings from Norwegian, Swedish, and Portuguese schools showing that boys tend to get higher grades as opposed to girls in PE (Ericsson and Cederberg, 2015; Lagestad, 2017; Marmeleira et al., 2020). It is important to note that these are average trends and that many girls during the adolescent period do improve their fitness and skill performance due to stimuli, experience, and practice gained from physical activities (Malina, 2014).

Despite neither motor competence nor physical fitness being explicitly expressed as assessment criteria in the Norwegian PE- curriculum, considerations from international and Scandinavian literature indicate that teachers' assessment practice and teaching content place value on these pupil-related factors. However, to the best of our knowledge, the potential association between these factors and academic achievement in PE has not previously been investigated. Hence, the current study aimed to investigate potential differences in motor competence and physical fitness between pupils with different academic achievements in PE attending lower secondary school (13–16 years). It was hypothesized that both (1) pupils with greater levels of motor competence would receive higher grades in PE compared to peers with lower levels of motor competence and (2) that pupils with greater levels of physical fitness would also receive higher grades in PE compared to peers with lower levels of physical fitness.

## Materials and Methods

### Participants

The sample consisted of 45 boys (14.44 ± 0.73 years) and 31 girls (14.41 ± 0.82 years) (*N* = 76) from grades 8 to 10 in one public lower secondary school located in a city in mid-Norway. A total of 30, 35, and 11 pupils represented grades 8, 9, and 10, respectively. All Norwegian public schools are co-educational and follow the same curricula, which was an inclusion criterion for invitation and participation. The sample's average grade was 5.02 (SD = 0.72) for boys and 4.81 (SD = 0.48) for girls. No pupils in this study had a grade lower than 3. Pupils were included if they followed normal school progression according to their year of birth and obtained a grade in PE.

Before data collection, all the participating pupils and their legal guardians signed written informed consent. It was made clear that participation was voluntary and that they could withdraw at any time. They were further informed that under no circumstances would their data be disclosed to anyone except the project leader. Ethical approval was obtained from the Norwegian Centre for Research Data (NSD) (#169464).

### Measurements

#### Grade

The participants' grade, as a proxy for academic achievement in PE, was collected after teachers' final end-of-year assessment in June 2020. This was ~3 months after collecting data on measures of motor competence and physical fitness. Grades, in whole numbers, are scored on a scale of 1–6 in the Norwegian school system, where a grade of 6 reflects the highest possible achievement (LOVDATA, 2006b). The national average grade for grade 10 pupils was 4.7 for both boys and girls in 2020 (Statistics Norway, 2020).

#### Motor Competence

To operationalize the concept of motor competence, the previously validated Test of Motor Competence (TMC) was used (Sigmundsson et al., 2016). The test consists of two fine motor tasks assessing manual dexterity and two gross motor tasks assessing dynamic balance. Scores for these motor tasks are measured in seconds, where a lower score indicates better performance. The four task items are as follows:

#### Fine Motor Tasks

1. Placing Bricks (PB). Pupils were to place 18 squared Duplo bricks on a 3 × 6 Duplo board while seated by a table. The bricks were placed in horizontal rows of three on the same side as the hand to be tested. The pupils were instructed to complete the task as fast as possible and hold the board firmly with the non-testing hand. Both hands were tested, and timing was stopped when the last brick was placed on the board. The score for each hand was summated. Before testing, they were given one practice run.
2. Building Bricks (BB). Pupils were to build a “tower” out of 12 squared Duplo bricks while seated by a table. The pupils were instructed to hold one brick in each hand, that they were not to rest their arms on the table, and that the bricks were to be held in the air at all times. They were further given a signal for when to start the test, and timing was stopped when they released contact with the last-placed brick. Only the dominant hand was tested. Before testing, they were given one practice run.

#### Gross Motor Tasks

3. Heal-to-toe walking (HTW). On a signal, pupils were to walk down a marked straight line (4.5 m) as fast as possible while placing their heel in contact with their toes for each step. The timing was stopped when the 4.5 m where completed.
4. Walking/running on slopes (W/R). Pupils were to walk/run as fast as possible in a figure of eight around two marked lines. The first line was placed 1 m from the starting point, while the second 5.5 m from the starting point. Each line was 1 m in width. The pupils were free to choose in which direction they wanted to start. Pupils were given a signal for when to start the tests, and timing was stopped when they arrived back at the starting point.

The test battery has previously shown an acceptable level of internal consistency among the standardized test items (*a* = 0.79) (Sigmundsson et al., 2016). Evaluated against the total score of the Movement Assessment Battery for Children-2 (MABC-2), a test widely used internationally, the total score TMC has a construct validity of.45 in a Norwegian adolescent sample (Sigmundsson et al., 2016). In the current study, a Principal Component Analysis (PCA) using an oblique rotation returned a one-factor solution with an eigenvalue of 2.34, accounting for 58.54% of the total variance, confirming the expected factor structure in this sample. The Cronbach's alpha was.76 for the standardized items in the current data.

#### Physical Fitness

Four tasks from the Test of Physical Fitness (TPF) (Fjørtoft et al., 2011) were selected for the assessment of lower body strength/power (standing broad jump), upper body strength/power (pushing medicine ball), speed (running 20 m as fast as possible), and endurance (reduced Cooper test). The task items are as follows:

#### Physical Fitness Tasks

1. Standing broad jump: The pupils start with their feet parallel and shoulder-width apart. On signal, they swing their arms backward and forward, jumping with both feet simultaneously as far forward as possible. The best score of two attempts was measured in centimeters from the starting to landing position. The pupils were instructed to stick their landing.
2. Pushing a medicine ball (2 kg): The pupils start with their feet parallel and shoulder-width apart, holding the medicine ball against their chest. On signal, they push the medicine ball from their chest as far forward as possible with both hands. Knee flexion was allowed. The best of 2 attempts was measured in centimeters from the starting to landing position of the medicine ball.
3. Running 20 m as fast as possible: The speed task was initiated 30 cm behind the speed gates to avoid false triggering. The pupils started with their lead leg on the line, the other in a backward position, and were instructed to avoid “rolling” backward when initiating the sprint. On signal, pupils were to run as fast as possible toward the finish line. The best of 2 attempts was measured in seconds.
4. Reduced Cooper test: The pupils were to run/walk as far as possible in 6 min on an indoor track (200 m in length). The achieved distance was measured in meters.

The TPF has previously been evaluated in Norwegian children, reporting an acceptable level of internal consistency (*a* = 0.93). The construct validity was assessed by comparing pupils' total test scores to that of their PE-teachers' evaluation or ranking of their physical fitness, resulting in a Spearman rho of.93 and.90 for boys and girls, respectively (Fjørtoft et al., 2011). The chosen items for this particular study have previously been used in a Norwegian adolescent population (Østerås et al., 2017). In this study, a PCA with an oblique rotation confirmed the expected one-factor solution with an eigenvalue of 2.79, accounting for 69.70% of the total variance. The Cronbach's alpha was 0.85 for the standardized items.

## Procedure

The assessment was conducted in an indoor sports hall for track and field during normal school hours. To avoid bringing too much physiological fatigue from one task to the other, all tasks were conducted in the order mentioned above, getting progressively harder. For instance, the reduced Cooper test was completed last, so that pupils did not bring any fatigue into the assessment of fine- and gross motor tasks, speed, and power/strength. Before the assessment, pupils conducted a standardized warm-up protocol for about 10 min to reduce potential injury risks. Pupils were given a short break between the warm-up and the initiation of assessment to reduce the heart rate to some extent before conducting tasks for fine motor skills. These tests were also organized so that all pupils were assessed individually, with trained practitioners stationed by a particular task. The individual task items were explained and demonstrated following the test manuals. Once the pupil completed one task, the trained practitioner recorded the result on a sheet, before he/she moved on to the next one. Enough time was given between the assessment of each pupil to avoid any communication between or feelings of discomfort with being observed by peers.

### Statistical Analysis

The data were analyzed using IBM SPSS version 27.0 (IBM Corp, 2020). The occurrence of missing values was low with 1.3% (running 20 m as fast as possible) and 3.9% (reduced Cooper test) missing. Little (1988) test of Missing Completely at Random (MCAR) indicated that data were missing completely at random (χ^2^ = 10.97, d.f. = 21, *p* =0.963). Missing values were treated by multiple imputation method (Schafer and Graham, 2002), which resulted in 5 iterations. The average value for these 5 iterations was used to replace the missing values for each individual case. Before the main analysis, data were tested for normality, skewness, and kurtosis. Age and all individual task items, except the reduced Cooper test, were transformed toward normality through a two-step approach (Templeton, 2011). This retains the original series mean and standard deviation, which helps the interpretation of results. To calculate a total score for both motor competence and physical fitness, individual task items were transformed into standardized scores (*z*-scores) from the mean of the whole sample. Before this, the item running 20 m as fast as possible was converted to 1/score such that higher scores equaled better performance similar to the other TPF items. The total test score for each pupil was calculated as the average *z*-score on all task items completed by the same pupil for both motor competence and physical fitness.

Gender and year of study (i.e., grades 8 vs. 9 vs. 10) specific differences in grade, age, total score motor competence, and physical fitness, and their respective task items were assessed by one-way ANOVA, separately. As there were no significant differences in the grade between boys and girls, or year of study in this sample, we did not further address these specific differences in the previously mentioned variables in relation to the PE grade. For the main analysis, a one-way ANOVA compared the mean value of total score motor competence and physical fitness, and their respective task items between groups of grades (i.e., 3 or 4, 5, and 6) for the total sample. Levene's test of Homogeneity of Variance suggested that the assumption of the equality of variance between groups was not met for some of the variables. Hence, Welch's F-statistics is reported for all variables in the analysis. Effect sizes were reported as partial eta-squared (η^2^) from the ANOVA F-statistics, and interpreted as small (0.01), medium (0.06), and large (0.14) (Cohen, 1988; Richardson, 2011). Due to heterogeneity between groups, the Games–Howell corrected pairwise comparisons were used *post-hoc* to locate differences between groups' mean values. The statistical significance level was set at *p* < 0.05.

## Results

### Differences in Grade, Age, Motor Competence, and Physical Fitness by Gender and Year of Study

Table 1 outlines the descriptive statistics (M ± SD) for grade, age, total score motor competence, and physical fitness, and their respective task items by gender. There were no significant differences in mean grade [Welch's *F*_(1,73.91)_ = 2.46, *p* = 0.121, η^2^ = 0.028] or age [Welch's *F*_(1,52.80)_ = 0.01, *p* = 0.912, η^2^ = 0.000] between boys and girls. However, boys portrayed a significantly better performance in the gross motor tasks HTW [Welch's *F*_(1,72.63)_ = 6.81, *p* =0.011, η^2^ =0.078] and W/R [Welch's *F*_(1,73.63)_ = 13.33, *p* = 0.000, η^2^ = 0.133], while no statistical differences were found for the fine motor tasks PB [Welch's *F*_(1,66.19)_ = 1.79, *p* = 0.185, η^2^ = 0.023] or BB [Welch's *F*_(1,73.42)_ = 1.15, *p* = 0.287, η^2^ = 0.014]. Additionally, the total score of motor competence [Welch's *F*_(1,73.99)_ = 1.29, *p* = 0.260, η^2^ = 0.015] was not statistically different between boys and girls. Regarding measures of physical fitness, boys both jumped [Welch's *F*_(1,73.86)_ = 6.32, *p* = 0.014, η^2^ =0.068] and pushed the medicine ball [Welch's *F*_(1,71.40)_ = 19.66, *p* = 0.000, η^2^ = 0.179] a longer distance compared to the girls. They also ran faster on the 20 m speed test [Welch's *F*_(1,71.30)_ = 17.39, *p* = 0.000, η^2^ = 0.180] and covered a longer distance in the reduced Cooper test [Welch's *F*_(1,73.57)_ = 20.61, *p* = 0.000, η^2^ = 0.200]. The total score of physical fitness was also significantly different in favor of the boys [Welch's *F*_(1,71.62)_ = 25.82, *p* = 0.000, η^2^ =0.223]. Regarding year of study (i.e., grades 8, 9, and 10), only the gross motor task HTW were significantly different between the study years [Welch's *F*_(2,31.74)_ = 4.67, *p* = 0.017, η^2^ = 0.105]. A Games–Howell corrected pairwise comparison revealed that pupils in the grade 9 performed the HTW significantly faster compared to grade 8 pupils [mean difference = −2.70, 95% CI (−5.116: −0.283)].

**Table 1:** Grade, age, and measures of physical fitness and motor competence by gender (*N* = 76).

**Variables**	**Girls (*N* = 31) (M/SD)**	**Boys (*N* = 45) (M/SD)**	**Total (*N* = 76) (M/SD)**
Grade	4.81 ± 0.48	5.02 ± 0.72	4.93 ± 0.64
Age (years)	14.41 ± 0.82 (14.48 ± 0.93)	14.44 ± 0.73 (14.46 ± 0.70)	14.43 ± 0.76 (14.47 ± 0.80)
Motor competence (z-score)	0.11 ± 0.58	−0.08 ± 0.86	–
Placing bricks (s)	46.94 ± 8.41	49.06 ± 8.40	48.20 ± 8.41
	(47.01 ± 8.52)	(49.72 ± 8.84)	(48.61 ± 8.76)
Building bricks (s)	12.41 ± 1.76	13.03 ± 2.82	12.78 ± 2.45
	(12.54 ± 2.11)	(13.14 ± 2.81)	(12.89 ± 2.55)
Heal to toe walking (s)^*^	11.78 ± 2.48	10.66 ± 4.70	11.12 ± 3.97
	(12.69 ± 3.42)	(10.36 ± 4.35)	(11.31 ± 4.13)
Walking/Running in slopes (s)^*^	4.81 ± 0.43	4.43 ± 0.55	4.59 ± 0.54
	(4.86 ± 0.38)	(4.45 ± 0.60)	(4.61 ± 0.56)
Physical fitness (z-score)^*^	−0.47 ±0.49	0.32 ± 0.87	–
Standing broad jump (cm)^*^	168.69 ± 20.77	183.37 ± 32.06	177.38 ± 28.78
	(169.40 ± 21.77)	(185.25 ± 33.22)	(178.78 ± 29.98)
Pushing medicine ball (cm)^*^	432.42 ± 50.22	526.76 ± 114.02	488.28 ± 103.99
	(438.48 ± 64.99)	(531.19 ± 116.35)	(493.38 ± 108.33)
20 m speed test (s)^*^	3.74 ± 0.27	3.53 ± 0.30	3.62 ± 0.30
	(3.79 ± 0.26)	(3.52 ± 0.31)	(3.63 ± 0.32)
Reduced Cooper test (m)^*^	1177.60 ± 150.74	1362.29 ± 203.71	1286.96 ± 204.41

*SD, standard deviation; M, mean*;

* *Significant difference between gender (p < 0.05). Values in parenthesis are mean and SD after transformation. For variables with parenthesis, all were transformed by the two-step process. A lower score on all items measured in seconds = better performance. A lower score on motor competence = better performance*.

### Motor Competence, Physical Fitness, and Academic Achievement in Physical Education

Table 2 outlines the descriptive statistics (M ± SD) for total score of motor competence and physical fitness, and their respective task items, for each group of grades. Neither total score of motor competence [Welch's *F*_(2,25.28)_ = 2.26, *p* = 0.125, η^2^ = 0.067] nor the TMC task items PB [Welch's *F*_(2,27.12)_ = 0.74, *p* = 0.486, η^2^ = 0.026], BB [Welch's *F*_(2,23.93)_ = 0.23, *p* = 0.793, η^2^ = 0.005] and HTW [Welch's *F*_(2,26.87)_ = 0.76, *p* = 0.477, η^2^ = 0.030] differed significantly between grades. In contrast, there was a significant and large difference between grades for the gross motor task W/R [Welch's *F*_(2,23.21)_ = 8.04, *p* = 0.002, η^2^ = 0.220]. According to the Games–Howell corrected pairwise comparisons, pupils with a grade of 6 [mean difference = −0.84, 95% CI (−1.359: −0.325)] and 5 [mean difference = −0.49, 95% CI (−0.936: −0.035)] completed the task significantly faster compared to pupils with a grade of 3 or 4.

**Table 2:** Measures of physical fitness and motor competence by grade (*N* = 76).

**Variables/Grade**	**3 or 4 (M/SD)**	**5 (M/SD)**	**6 (M/SD)**	**Total (M/SD)**
Motor competence (z-score)	0.34 ± 0.1.06	−0.04 ± 0.67	−0.28 ± 0.50	–
Placing bricks (s)	50.50 ± 11.91	47.93 ± 7.74	46.21 ± 4.59	48.20 ± 8.41
	(51.25 ± 11.44)	(48.08 ± 8.39)	(47.22 ± 5.47)	(48.61 ± 8.76)
Building bricks (s)	12.91 ± 3.84	12.82 ± 2.00	12.41 ± 1.91	12.78 ± 2.45
	(12.77 ± 3.27)	(13.03 ± 2.41)	(12.54 ± 2.20)	(12.90 ± 2.55)
Heal to toe walking (s)	12.97 ± 7.27	10.69 ± 2.47	10.39 ± 1.67	11.12 ± 3.97
	(12.69 ± 5.37)	(10.97 ± 3.95)	(10.86 ± 2.64)	(11.31 ± 4.13)
Walking/running in slopes (s)^*^	5.04 ± 0.66	4.54 ± 0.42	4.19 ± 0.35	4.59 ± 0.54
	(5.05 ± 0.66)	(4.57 ± 0.45)	(4.21 ± 0.44)	(4.61 ± 0.56)
Physical fitness (z-score)^*^	−0.52 ± 0.86	−0.03 ± 0.66	0.79 ± 0.88	–
Standing broad jump (cm)^*^	156.69 ± 34.92	176.07 ± 21.96	206.17 ± 23.59	177.38 ± 28.78
	(161.47 ± 30.59)	(176.57 ± 23.56)	(210.73 ± 30.80)	(178.78 ± 29.98)
Pushing medicine ball (cm)	468.56 ± 122.64	475.77 ± 82.95	564.58 ± 127.39	488.28 ± 103.99
	(475.72 ± 144.91)	(484.22 ± 81.95)	(553.52 ± 133.85)	(493.38 ± 108.33)
20 m speed test (s)^*^	3.79 ± 0.35	3.60 ± 0.28	3.44 ± 0.21	3.62 ± 0.30
	(3.80 ± 0.31)	(3.62 ± 0.30)	(3.44 ± 0.27)	(3.63 ± 0.32)
Reduced Cooper test (m)^*^	1,121.63 ± 168.23	1,296.12 ± 178.33	1,470.75 ± 182.85	1,286.96 ± 204.41

*SD, standard deviation; M, mean*;

* *Significant difference between groups of grades by ANOVA (p < 0.05). Values in parenthesis are mean and SD after transformation. For variables with parenthesis, all were transformed by the two-step process. A lower score on all items measured in seconds = better performance. A lower score on Motor competence = better performance*.

Except for pushing medicine ball [Welch's *F*_(2,20.02)_ = 1.49, *p* = 0.249, η^2^ = 0.060], total score of physical fitness [Welch's *F*_(2,21.76)_ = 7.49, *p* = 0.003, η^2^ = 0.226] and the remaining task items standing broad jump (Welch's *F*(2, 21.77) = 8.93, *p* = 0.001, η^2^ = 0.256], running 20 m as fast as possible [Welch's *F*_(2,25.20)_ = 5.12, *p* = 0.014, η^2^ =0.118], and reduced Cooper test [Welch's *F*_(2,24.59)_ = 13.45, *p* = 0.000, η^2^ = 0.270] were significantly different between grades with moderate-to-large effect sizes. The Games–Howell corrected pairwise comparisons revealed that pupils with a grade of 6 had a significantly better total score of physical fitness compared to pupils with grades of 5 [mean difference = 0.81, 95% CI (0.107: 1.523)] and 3 or 4 [mean difference = 1.30, 95% CI (0.472: 2.137)]. They also jumped longer compared to pupils with a grade of 5 [mean difference = 34.16, 95% CI (9.340: 58.985)] and 3 or 4 [mean difference = 49.26, 95% CI (19.952: 78.559)], and ran faster than pupils with a grade of 3 or 4 [mean difference = −0.34, 95% CI (−0.635: −0.083)]. In regard to the reduced Cooper test, there was a significant increase in performance between groups of grades where pupils receiving a grade of 6 covered a longer distance compared to pupils receiving a grade of 5 [mean difference = 174.63, 95% CI (23.67: 325.60)] and 3 or 4 [mean difference = 349.13, 95% CI (179.95: 518.30)], while those pupils with a grade of 5 covered a longer distance than those with a grade of 3 or 4 [mean difference = 174.49, 95% CI (52.27: 296.71)].

## Discussion

The main aim of the study was to investigate potential differences in motor competence and physical fitness between 13- and 16-year-old school children with different academic achievements in PE. The main findings indicated that, except for W/R, neither the total score of motor competence nor the other respective task items of TMC were significantly different between pupils receiving a grade of 3 or 4, 5, or 6. Furthermore, with the exception of pushing a medicine ball as far as possible, findings indicated moderate-to-large differences between achievement groups in a total score of physical fitness and the other task items of physical fitness, in which pupils with a higher academic achievement were generally more physically fit as opposed to pupils with lower academic achievement in PE.

### Differences in Grade, Age, Motor Competence, and Physical Fitness by Gender and Year of Study

In the current sample, there were no significant differences between the adolescent boys and girls in academic achievement in PE. This might be a reasonable finding as the national average academic achievement corresponds with the current sample's average for both boys and girls. Despite the lack of any gender-related differences, the boys significantly outperformed the girls in both gross motor tasks and all aspects of physical fitness. The gross motor task items HTW and W/R aim to measure aspects of coordination, speed, change of direction, and dynamic balance (Pasanen et al., 2009; Sigmundsson et al., 2016). Previous research has, in contrast to our findings, reported no gender-related differences in gross motor tasks such as hop, side gallop, vertical jump, and sprint (Okely et al., 2004; Barnett et al., 2010). This discrepancy could potentially be explained by a difference in assessment procedures, that is, the use of process or product-oriented measures. Both Barnett et al. (2010) and Okely et al. (2004) conducted a qualitative assessment of the aforementioned motor tasks, assessing how well the tasks were executed, while the current study assessed the gross motor tasks in a quantitative manner (i.e., time and distance). As the execution of motor skills may additionally require components of physical fitness (Gallahue and Ozmun, 2006; Vandendriessche et al., 2011), adolescent boys may have an advantage in quantitative assessment procedures due to maturational differences. In particular, during the adolescent growth spurt, boys tend to develop greater levels of strength, power, speed, and endurance compared to girls (Malina et al., 2004), which may also explain why boys in the present study had a better total score of physical fitness as well as outperforming the girls on each of the individual task items for physical fitness. Due to the lack of a statistically significant difference in achievement between genders, no further gender-specific differences in motor competence or physical fitness related to achievement were examined in subsequent analysis.

### Motor Competence, Physical Fitness, and Academic Achievement in Physical Education

As opposed to our hypothesis that pupils with greater levels of motor competence would receive higher academic achievement in PE, no difference between groups of grades in the total score of motor competence, fine motor tasks, or the gross motor task HTW was found. The TMC-battery is aimed at capturing individuals' motor competence across the life span, with adequate performance levels of the respective task items that are important for individuals' ability to carry out and participate in everyday activities (Sigmundsson et al., 2016). As such, one could argue that the test battery has a more general approach to the concept of motor competence and that future studies should instead address more specific motor skills in the context of PE. For example, when ball sports are to a large extent included in Norwegian PE (Moen et al., 2018), pupils' ability to control objects (e.g., catching, throwing) could potentially influence their performance and achievement within the subject when PE-teachers seem to place value on sport-specific skills in assessment (Aasland et al., 2020). The present study's measurement of fine motor performance, however, assessed pupils' level of manual dexterity (Sigmundsson et al., 2016). Even though this entails hand-eye coordination, among other aspects, these task items could have failed to adequately replicate the movement constraints in PE (e.g., catching, throwing). Motor tasks are generally considered highly specific with low inter-correlations (Haga et al., 2008; Giboin et al., 2015; Sigmundsson et al., 2021), thus highlighting the need for addressing other dimensions of motor behavior and their potential relationship with academic achievement in PE.

Opposite to the above-mentioned findings, the item W/R was the only (gross) motor task significantly different between achievement groups. The lowest achievement group completed the task considerably slower as opposed to pupils with higher achievement. It is argued that this task captures an individual's ability to accelerate, decelerate, and change direction (Pasanen et al., 2009), placing demands on fitness aspects such as strength, power, and speed (Gísladóttir et al., 2019). Consequently, this gross motor task could have explained differences between achievement groups through its physical fitness component.

Confirming our hypothesis, the current study found higher achieving pupils to have greater levels of overall physical fitness and outperform the lower achieving pupils in three of the four physical fitness task items (i.e., standing broad jump, running 20 m as fast as possible, and reduced Cooper test). Hence, these findings support research indicating a sport and exercise discourse within PE (Redelius et al., 2009; Kirk, 2010; López-Pastor et al., 2013; Aasland et al., 2017, 2020; Erdvik, 2020). These findings might be expected given that there have traditionally been, and still are, many PE-teachers who apply physical fitness testing to assess pupils' achievement level within the subject (Cale and Harris, 2009; López-Pastor et al., 2013; Cale et al., 2014; Alfrey and Gard, 2019; Marmeleira et al., 2020). This assessment practice seems to endure among Norwegian PE-teachers (Prøitz and Borgen, 2010; Moen et al., 2018; Aasland et al., 2020), despite physical fitness testing as a means for assessing pupils in a norm-references manner having been referenced as problematic and potentially a direct violation of the Regulations Pursuant to the Education Act (LOVDATA, 2006a; The Norwegian Directorate for Education and Training, 2015b). It should be acknowledged, however, that due to Norway's highly decentralized education system, where teachers experience considerable autonomy in their teaching and assessment practices (Tveit, 2014; Leirhaug and Annerstedt, 2016), there may be discrepancies between schools in the use of fitness testing. Even so, pupils with greater levels of physical fitness may still achieve better assessment based on their ability of high exertion. For instance, Norwegian PE-teachers are reported to interpret the effort criterion as pupils' ability in working physically hard, with their assessment based on the visible behavior within the activity which is taught (Aasland and Engelsrud, 2017). When the subject then is dominated by sports and fitness training (Moen et al., 2018), pupils with greater levels of physical fitness may not only have the capacity to exert greater levels of effort in line with the teachers' understanding of the effort criterion, but also be given the possibility to, and be rewarded for, the ability to express strength, power, and endurance at a higher level compared to their peers. Furthermore, a recent study revealed that Norwegian pupils with high and low levels of physical fitness (i.e., measured by oxygen uptake) experienced considerable discrepancy in the quantity of assessment received from their PE-teachers, with the less physically fit pupils experiencing less attention and feedback (Lyngstad et al., 2020). Potentially, this inequality may further reinforce individual differences in achievement based on their physical fitness levels.

## Methodological Considerations

Some methodological limitations and strengths need to be acknowledged, which hopefully can inform further studies. First, it is important to note that the cross-sectional design and the moderate sample size do not warrant any causal conclusions. Additionally, the objective assessment of motor competence and physical fitness could have caused a selection bias. Potentially, pupils more confident in their motor competence and physical fitness chose to participate. However, the current sample's high average academic achievements in PE were in proximity with the national average for grade 10 pupils. This suggests that the sample had some resemblance to the general population in the Norwegian lower secondary school level. Before and during data collection, we ensured that all testing was conducted in a safe and private environment so that pupils avoided being observed by their respective PE-teachers or their peers. Second, as highlighted in the constraints-led theoretical perspective, the grade in PE could be viewed as a result of the dynamic and mutually influential relationship between individual, task, and environmental constraints. In the current study, the rationale for investigating motor competence and physical fitness is rooted in the current knowledge regarding teaching content, discourses, and teachers' assessment practices (i.e., task/environmental constraints). All these constraints are not captured in the current study but might influence which pupil-related factors are of relevance in understanding their academic achievement within PE. Third, as motor performance is deemed as being highly task specific, the current task items for fine motor assessment may have failed to adequately replicate or represent movement activities within PE, and as such explain the lack of finding any significant differences between achievement groups.

## Conclusion

To the authors' knowledge, the current study is first in identifying differences in physical fitness, and one component of gross motor competence, between pupils with different academic achievements in PE. Regarding the hypothesis of pupils with greater levels of motor competence receiving higher grades in PE, only the gross motor task W/R were significantly different in favor of pupils with the highest academic achievement level. In general, this implies that the first hypothesis is rejected. In contrast, the results confirmed the second hypothesis expecting more physically fit pupils to receive higher grades as opposed to their less physically fit peers. Collectively, these results offer insight into individual constraints with the potential to influence pupils' academic achievement in PE. More specifically, PE-teachers should acknowledge individual differences in physical fitness levels in their teaching and assessment practice, addressing systematic bias in their chosen teaching content and assessment criteria.

## Data Availability Statement

The original contributions presented in the study are included in the article/supplementary material, further inquiries can be directed to the corresponding author.

## Ethics Statement

The studies involving human participants were reviewed and approved by Norwegian Centre for Research Data (NSD). Written informed consent to participate in this study were provided by the participants and their legal guardians/next of kin.

## Author Contributions

RVH, HL, HS, and MH contributed to the conception and design of the study. RVH collected the data. RVH and HL analyzed the data. RVH wrote the first draft of the manuscript. All authors contributed to manuscript revision and read and approved the submitted version.

## Conflict of Interest

The authors declare that the research was conducted in the absence of any commercial or financial relationships that could be construed as a potential conflict of interest.

## Publisher's Note

All claims expressed in this article are solely those of the authors and do not necessarily represent those of their affiliated organizations, or those of the publisher, the editors and the reviewers. Any product that may be evaluated in this article, or claim that may be made by its manufacturer, is not guaranteed or endorsed by the publisher.

## References

[B1] The Norwegian Directorate for Education and Training. (2015a). Curriculum for Physical Education (KRO1-04) (2015). Available online at: https://www.udir.no/kl06/KRO1-04?lplang=http://data.udir.no/kl06/eng (accessed August 20, 2021).

[B2] The Norwegian Directorate for Education and Training. (2015b). Endringer i faget kroppsøving (Udir-8-2012). Available online at: https://www.udir.no/regelverkstolkninger/opplaring/Innhold-i-opplaringen/Udir-8-2012/ (accessed August 20, 2021).

[B3] Aasland E. Engelsrud G. (2017). Det er lett å se hvem av dere som har god innsats. Om elevers innsats og lærerens blikk i kroppsøving. J. Res. Arts Sports Educ. 1, 5–17. 10.23865/jased.v1.889

[B4] Aasland E. Walseth K. Engelsrud G. (2017). The changing value of vigorous activity and the paradox of utilising exercise as punishment in physical education. Phys. Educ. Sport Pedagogy. 22, 490–501. 10.1080/17408989.2016.1268590

[B5] Aasland E. Walseth K. Engelsrud G. (2020). The constitution of the ‘able’ and ‘less able’ student in physical education in Norway. Sport Educ. Soc. 25, 479–92. 10.1080/13573322.2019.1622521

[B6] Adolph K. E. Hoch J. E. (2019). Motor development: embodied, embedded, enculturated, and enabling. Annu. Rev. Psychol. 70, 141–64. 10.1146/annurev-psych-010418-102836 30256718 PMC6320716

[B7] Alfrey L. Gard M. (2019). Figuring out the prevalence of fitness testing in physical education: a figurational analysis. Eur. Phys. Educ. Rev. 25, 187–202. 10.1177/1356336X17715361

[B8] Annerstedt C. Larsson S. (2010). ‘I have my own picture of what the demands are…’: grading in Swedish PEH—problems of validity, comparability and fairness. Eur. Phy Educ Rev. 16, 97–115. 10.1177/1356336X10381299

[B9] Arnesen T. Nilsen A. K. Leirhaug P. E. (2013). ‘Den læreplanen som ikkje kan tilpassast mi undervisning, finst ikkje ’: Vurdering og undervisning i kroppsøving etter kunnskapsløftet. Tidsskriftet FOU i Praksis. 7, 9–32.

[B10] Barnett L. M. Van Beurden E. Morgan P. J. Brooks L. O. Beard J. R. (2010). Gender differences in motor skill proficiency from childhood to adolescence: a longitudinal study. Res. Q. Exerc. Sport. 81, 162–70. 10.5641/027013610X13088554297116 20527301

[B11] Baxter-Jones A. D. (1995). Growth and development of young athletes. Should competition levels be age related? Sports Med. 20, 59–64. 10.2165/00007256-199520020-00001 7481282

[B12] Borghouts L. B. Slingerland M. Haerens L. (2017). Assessment quality and practice in secondary PE in the Netherlands. Phys. Educ. Sport Pedagogy. 22, 473–89. 10.1080/17408989.2016.1241226

[B13] Burton A. W. Miller D. E. (1998) Movement Skill Assessment. Champaign, IL: Human Kinetics.

[B14] Cale L. Harris J. (2009). Fitness testing in physical education—a misdirected effort in promoting healthy lifestyles and physical activity? Phys. Educ. Sport Pedagogy. 14, 89–108. 10.1080/17408980701345782

[B15] Cale L. Harris J. Chen M. H. (2014). Monitoring health, activity and fitness in physical education: its current and future state of health. Sport Educ. Soc. 19, 376–97. 10.1080/13573322.2012.681298

[B16] Caspersen C. J. Powell K. E. Christenson G. M. (1985). Physical activity, exercise, and physical fitness: definitions and distinctions for health-related research. Public Health Rep. 100, 126–31. 3920711 PMC1424733

[B17] Cobley S. McKenna J. Baker J. Wattie N. (2009). How pervasive are relative age effects in secondary school education? J Educ Psychol. 101, 520–8. 10.1037/a0013845

[B18] Cohen J. (1988). Statistical Power Analysis for the Behavioral Sciences. New York, NY: Routledge.

[B19] Davids K. Button C. Bennett S. (2008) Dynamics of Skill Acquisition: A Constraints-Led Approach. Champaign, IL: Human Kinetics.

[B20] Erdvik I. B. (2020). Physical education as a developmental asset in the everyday life of adolescents. A relational approach to the study of basic need satisfaction in PE and global self-worth development. [doctoral thesis]. Oslo, Norway: Norwegian School of Sport Sciences

[B21] Ericsson I. Cederberg M. (2015). Physical activity and school performance: a survey among students not qualified for upper secondary school. Phys Educ Sport Pedagogy. 20, 45–66. 10.1080/17408989.2013.788146

[B22] Fjørtoft I. Pedersen A. V. Sigmundsson H. Vereijken B. (2011). Measuring physical fitness in children who are 5 to 12 years old with a test battery that is functional and easy to administer. Phys Ther. 91, 1087–95. 10.2522/ptj.20090350 21616933

[B23] Fransen J. Deprez D. Pion J. Tallir I. B. D'Hondt E. Vaeyens R. (2014). Changes in physical fitness and sports participation among children with different levels of motor competence: a 2-year longitudinal study. Pediatr Exerc Sci. 26, 11–21. 10.1123/pes.2013-0005 24018944

[B24] Gallahue D. L. Ozmun J. C. (2006) Understanding Motor Development: Infants, Children, Adolescents, Adults. New York, NY: McGraw Hill.

[B25] Giboin L. S. Gruber M. Kramer A. (2015). Task-specificity of balance training. Hum. Mov. Sci. 44, 22–31. 10.1016/j.humov.2015.08.012 26298214

[B26] Gísladóttir T. Haga M. Sigmundsson S. (2019). Motor competence in adolescents: Exploring associations with physical fitness. Sports. 7:176. 10.3390/sports7070176 31330808 PMC6681283

[B27] Haga M. (2009). Physical fitness in children with high motor competence is different from that in children with low motor competence. Phys Ther. 89, 1089–97. 10.2522/ptj.20090052 19679648

[B28] Haga M. Pedersen A. V. Sigmundsson H. (2008). Interrelationship among selected measures of motor skills. Child Care Health Dev. 34, 245–8. 10.1111/j.1365-2214.2007.00793.x 18028473

[B29] Hay P. J. Macdonald D. (2010). Evidence for the social construction of ability in physical education. Sport Educ. Soc. 15, 1–18. 10.1080/1357332090321707525141091

[B30] Henderson E. Sugden A. (1992) Movement Assessment Battery for Children-2. London, UK: Pearson Education.

[B31] Holfelder B. Schott N. (2014). Relationship of fundamental movement skills and physical activity in children and adolescents: a systematic review. Psychol. Sport Exerc. 15, 382–91. 10.1016/j.psychsport.2014.03.005

[B32] IBM Corp. (2020). SPSS. Statistics for Windows. Version 270. Armonk, NY: IBM Corp.

[B33] Kirk D. (2010). Physical Education Futures. London, UK: Routledge.

[B34] Lagestad P. (2017). Er gutter bedre enn jenter i kroppsøving? En studie av jenter og gutters kroppsøvingskarakterer i den videregående skolen. Acta Didact Nor. 11:5. 10.5617/adno.2609

[B35] Leirhaug P. E. (2016). Exploring the relationship between student grades and assessment for learning in Norwegian physical education. Eur. Phys. Educ. Rev. 22, 298–314. 10.1177/1356336X15606473

[B36] Leirhaug P. E. Annerstedt C. (2016). Assessing with new eyes? Assessment for learning in Norwegian physical education. Phys Educ Sport Pedagogy. 21, 616–31. 10.1080/17408989.2015.1095871

[B37] Leirhaug P. E. MacPhail A. (2015). ‘It's the other assessment that is the key’: three Norwegian physical education teachers' engagement (or not) with assessment for learning. Sport Educ. Soc. 20, 624–40. 10.1080/13573322.2014.975113

[B38] Little R. J. A. (1988). A test of missing completely at random for multivariate data with missing values. J Am Stat Assoc. 83, 1198–202. 10.1080/01621459.1988.10478722

[B39] López-Pastor V. M. Kirk D. Lorente-Catalán E. MacPhail A. Macdonald D. (2013). Alternative assessment in physical education: a review of international literature. Sport Educ Soc. 18, 57–76. 10.1080/13573322.2012.713860

[B40] LOVDATA. (2006a). Regulations Pursuant to the Education Act. Regulations to the Education Act (FOR-2006-06-23-724), § 3-3. Norway: LOVDATA

[B41] LOVDATA. (2006b). Regulations Pursuant to the Education Act. Regulations to the Education Act (FOR-2006-06-23-724), § 3-5. Norway: LOVDATA.

[B42] Lyngstad I. Bjerke Ø. Bang K. M. Lagestad P. (2020). Norwegian upper secondary students' experiences of their teachers' assessment of and for learning in physical education: examining how assessment is interpreted by students of different physical abilities. Sport Educ. Soc. 2020:1–12. 10.1080/13573322.2020.1842728

[B43] Malina R. M. (2014). Top 10 research questions related to growth and maturation of relevance to physical activity, performance, and fitness. Res Q Exerc Sport. 85, 157–73. 10.1080/02701367.2014.897592 25098012

[B44] Malina R. M. Bouchard C. Bar-Or O. (2004). Growth, Maturation, and Physical Activity. Champaign, IL: Human Kinetics.

[B45] Marmeleira J. Folgado H. Guardado I. M. Batalha N. (2020). Grading in Portuguese secondary school physical education: assessment parameters, gender differences and associations with academic achievement. Phys. Educ. Sport Pedagogy. 25, 119–36. 10.1080/17408989.2019.1692807

[B46] Moen K. M. Westlie K. Bjørke L. Brattli V. H. (2018). Når Ambisjon Møter Tradisjon: En Nasjonal Kartleggingsstudie av Kroppsøvingsfaget i Grunnskolen (5.−10. trinn). Elverum, Norway: Høgskolen i Innlandet.

[B47] Newell K. M. (1986) Constraints on the development of coordination, in Motor Development in Children: Aspects of Coordination and Control, eds Wade M. Whiting H. (Dordrecht, Netherlands: Martinus Nijhoff), 341–360.

[B48] Okely A. D. Booth M. L. Chey T. (2004). Relationships between body composition and fundamental movement skills among children and adolescents. Res. Q. Exerc. Sport. 75, 238–247. 10.1080/02701367.2004.10609157 15487288

[B49] Ortega F. B. Ruiz J. R. Castillo M. J. Sjöström M. (2008). Physical fitness in childhood and adolescence: a powerful marker of health. Int. J. Obes. 32, 1–11. 10.1038/sj.ijo.0803774 18043605

[B50] Østerås B. Sigmundsson H. Haga M. (2017). Physical fitness levels do not affect stress levels in a sample of Norwegian adolescents. Front. Psychol. 8:2176. 10.3389/fpsyg.2017.02176 29326625 PMC5733357

[B51] Pasanen K. Parkkari J. Pasanen M. Kannus P. (2009). Effect of a neuromuscular warm-up programme on muscle power, balance, speed and agility: a randomised controlled study. Br. J. Sports Med. 43, 1073–8. 10.1136/bjsm.2009.061747 19622526

[B52] Philippaerts R. M. Vaeyens R. Janssens M. Van Renterghem B. Matthys D. Craen R. (2006). The relationship between peak height velocity and physical performance in youth soccer players. J. Sports Sci. 24, 221–30. 10.1080/02640410500189371 16368632

[B53] Prøitz T. S. Borgen J. S. (2010). Rettferdig Standpunktvurdering – Det (U)Muliges Kunst? Læreres Setting av Standpunktkarakter i Fem Fag i Grunnopplæringen. Report 16/2010. Oslo, Norway: NIFU STEP.

[B54] Redelius K. Fagrell B. Larsson H. (2009). Symbolic capital in physical education and health: to be, to do or to know? That is the gendered question. Sport Educ. Soc. 14, 245–60. 10.1080/13573320902809195

[B55] Richardson J. T. E. (2011). Eta squared and partial eta squared as measures of effect size in educational research. Educ. Res. Rev. 6, 135–47. 10.1016/j.edurev.2010.12.001

[B56] Schafer J. L. Graham J. W. (2002). Missing data: our view of the state of the art. Psychol. Methods. 7, 147–77. 10.1037/1082-989X.7.2.147 12090408

[B57] Sigmundsson H. Lorås H. Haga M. (2016). Assessment of motor competence across the life span: aspects of reliability and validity of a new test battery. New York: SAGE Open.

[B58] Sigmundsson H. Newell K. M. Polman R. Haga M. (2021). Exploration of the specificity of motor skills hypothesis in 7-8 year old primary school children: exploring the relationship between 12 different motor skills from two different motor competence test batteries. Front. Psychol. 12, 631175. 10.3389/fpsyg.2021.631175 34220608 PMC8249579

[B59] Sigmundsson H. Trana L. Polman R. Haga M. (2017). What is trained develops! Theoretical perspective on skill learning. Sports. 5:38. 10.3390/sports5020038 29910400 PMC5968992

[B60] Statistics Norway. (2020). Marks, lower secondary school (2020). https://www.ssb.no/en/utdanning/statistikker/kargrs (accessed August 20, 2021).

[B61] Svenneberg L. Meckbach J. Redelius K. E. (2014). Teachers “gut feelings”: an attempt to verbalise and discuss teachers' internalised grading criteria. Eur. Phys. Educ. Rev. 20, 199–214. 10.1177/1356336X13517437

[B62] Templeton G. F. (2011). A two-step approach for transforming continuous variables to normal: implications and recommendations for IS research. Commun. Assoc. Inf. Syst. 28, 41–58. 10.17705/1CAIS.02804

[B63] Tveit S. (2014). Educational assessment in Norway. Assess. Educ. Princ. Policy Pract. 21, 221–37. 10.1080/0969594X.2013.830079

[B64] Utesch T. Bardid F. (2019) Motor competence, in Dictionary of Sport Psychology: Sport, Exercise, Performing Arts 1st ed, eds Hackfort D. Schinke R. Strauss B. (Berlin/Heidelberg: Elsevier).

[B65] Vandendriessche J. B. Vandorpe B. Coelho-e-Silva M. J. Vaeyens R. Lenoir M. Lefevre J.. (2011). Multivariate association among morphology, fitness, and motor coordination characteristics in boys age 7 to 11. Pediatr. Exerc. Sci. 23, 504–20. 10.1123/pes.23.4.504 22109777

